# Progress in the Study of Chemical Constituents of *Actaea cimicifuga* and *Actaea erythrocarpa* and Their Biological Potential

**DOI:** 10.3390/ijms26104768

**Published:** 2025-05-16

**Authors:** Andrey S. Erst, Natalia V. Petrova, Alexander A. Chernonosov, Olga A. Kaidash, Vladimir V. Sheikin, Tatiana V. Leonova, Tatiana M. Shaldaeva, Anastasiia S. Gusar, Vladimir V. Koval, Elena V. Udut, Kunli Xiang, Yuan-Yuan Ling, Wei Wang, Vera A. Kostikova

**Affiliations:** 1 Central Siberian Botanical Garden, Siberian Branch of Russian Academy of Sciences (SB RAS), Novosibirsk 630090, Russia; tshaldaeva@yandex.ru (T.M.S.); gusara663@gmail.com (A.S.G.); serebryakova-va@yandex.ru (V.A.K.); 2 Komarov Botanical Institute of Russian Academy of Sciences, St. Petersburg 197022, Russia; 3 Institute of Chemical Biology and Fundamental Medicine, Siberian Branch of Russian Academy of Sciences (SB RAS), Novosibirsk 630090, Russia; alexander.chernonosov@niboch.nsc.ru (A.A.C.); koval@niboch.nsc.ru (V.V.K.); 4 Central Research Laboratory, Siberian State Medical University, Tomsk 634050, Russia; kaidash_2011@mail.ru (O.A.K.); sheykinvv@gmail.com (V.V.S.); evu8@mail.ru (E.V.U.); 5 Institute of Natural Sciences, Department of Biology, N.F. Katanov Khakass State University, Abakan 655517, Russia; geoides76@mail.ru; 6 State Key Laboratory of Plant Diversity and Prominent Crops, Institute of Botany, Chinese Academy of Sciences, Beijing 100093, China; kunlixiang@ibcas.ac.cn (K.X.); lingyuanyuan@ibcas.ac.cn (Y.-Y.L.); wangwei1127@ibcas.ac.cn (W.W.)

**Keywords:** *Actaea*, antioxidant and antitumor activity, biologically active substances, liquid chromatography–high-resolution mass spectrometry (LC-HRMS), high-performance liquid chromatography (HPLC)

## Abstract

For the first time, hydroethanolic extracts from *Actaea cimicifuga* and *Actaea erythrocarpa* were analyzed using LC-HRMS, HPLC, and spectrometry in this study. Extracts from the above-ground parts of *Actaea* species exhibited higher concentrations of saponins (up to 248 mg/g of DE), coumarins (up to 162 mg/g of DE), flavonols (up to 32 mg/g of DE), and catechins (up to 11 mg/g of DE) compared to extracts from the underground parts. The concentrations of phenolic acids (up to 112 mg/g of DE) and tannins (up to 202 mg/g of DE) in the underground parts were comparable to or even higher than those in the above-ground parts of the two analyzed species. The concentration of the main metabolites detected was higher in the extract of *A. erythrocarpa* than that of *A. cimicifuga*. The metabolite profile of the extracts from both species showed 66 compounds, including chromones, coumarins, phenolic and nitrogenous compounds, fatty acids, and triterpenes. The HPLC analysis of the four extracts revealed that the concentration of caffeic acid (0.74 mg/g of the dry extract [DE]) was the highest in the extract from the underground part of *A. erythrocarpa*, whereas the extract from the above-ground part of this species showed the highest levels of ferulic (1.16 mg/g of DE) and isoferulic acids (1.49 mg/g of DE) and of hyperoside (13.05 mg/g of DE). The study of biological activity showed that *A. erythrocarpa* is most promising for further research, with the highest antioxidant activity found in the underground parts of this species (IC_50_ = 79.7 μg/mL) compared to the above-ground parts (IC_50_ = 85.8 μg/mL). In addition, the extract from the above-ground part of *A. erythrocarpa* was found to exhibit the greatest cytotoxic activity among the studied specimens against 3T3-L1, HepG2, and MDA-MB-231 cells.

## 1. Introduction

The genus *Actaea* is an herbaceous genus from the tribe Cimicifugeae Torrey et Gray of the family Ranunculaceae Juss. *Actaea* includes ca. 35 species in the Northern Hemisphere, except for *A. japonica*, whose geographic range extends to subtropical evergreen broad-leaved forests [1]. The fleshy berries of *Actaea* and the dry follicles of *Cimicifuga* have been used to distinguish this species [2,3]. On the basis of two molecular and morphological datasets, some researchers [3] further validated this approach and redefined *Actaea* to include *Cimicifuga* and *Souliea*, and this taxonomy has been accepted by subsequent investigators [4].

Interest in species of the genus *Actaea* has persisted for centuries. *A. racemosa* L. (syn. *Cimicifuga racemosa* L.) has become the most famous: its raw materials and derived products are among the 10 most in-demand plants in the USA [5]. Initially, *A. racemosa* was used not only as a food plant; Native Americans and colonists also employed it as a remedy for menstrual aberrations, malaria, kidney dysfunction, and other health problems [6]. Such uses have been validated scientifically but much later. Research in *A. racemosa*, which is still in full swing, began in the 1960s–1970s; it has led to the discovery of ~300 compounds of various classes. The main active ingredients are triterpene glycosides (such as actein, 27-deoxyactein, cimicifugoside, etc.), phenolic compounds (for example, isoferulic acid and its derivatives), quinolizidine alkaloids, and some other phytochemical components of *A. racemosa*, which are the subject of several reviews [7,8,9], including pharmacological experiments on total extracts, fractions, and individual components of this plant.

Three *Actaea* species [*A. dahurica* Franch., *A. cimicifuga* (Schipcz) J. Compton, and *A. heracleifolia* (Kom.) J. Compton] are common in traditional Chinese medicine and are included in the Chinese pharmacopeia [10], whereas the Japanese pharmacopeia, aside from the above-mentioned species, also includes *A. simplex* Wormsk. ex. Prantl. [11]. In Europe, *A. spicata* L. has been traditionally utilized as an insecticide [12]. In general, it has been revealed that extracts from plants of the genus *Actaea* possess antiosteoporosis, antiviral, antioxidant, antitumor, antiangiogenic, and other types of activity and reduce symptoms of climacteric syndrome in women [13,14,15]. Therefore, the search for biologically active substances with pronounced pharmacological activity among *Actaea* species is promising. This is particularly true for relatively little-studied *Actaea* species such as *A. cimicifuga* (Schipcz), J. Compton., and *A. erythrocarpa* (Fisch.) Freyn.

Until recently, *A. cimicifuga* was classified as *Cimicifuga foetida*; according to the newest data, this taxon is included in the section *Cimicifuga* (L. ex Wernisch.) DC. of the genus *Actaea. A. erythrocarpa* is a member of the section *Actaea* and, according to the taxonomic treatment of the genus [3], is a synonym of *A. rubra* (Aiton) Willd. Nonetheless, according to our earlier data, the American species *A. rubr*a is not found in Eurasia and is represented by another species: *A. erythrocarpa* [16]. In the present work, we agree with and use the generally accepted names of the species: *A. erythrocarpa* and *A. cimicifuga* [17].

The component composition of *A. erythrocarpa* has not been previously studied; *A. cimicifuga* is known to accumulate a complex of terpenes belonging to monoterpenes and a series of 9,19-cycloartane triterpenes and their glycosides of various structures, most of which demonstrate promising biological activity [15]. The aim of the present study was to investigate biologically active substances and antioxidant and antitumor activity in *A. cimicifuga* and *A. erythrocarpa* growing in Siberia (Russia).

## 2. Results and Discussion

### 2.1. Levels of Biologically Active Substances in Extracts from A. cimicifuga and A. erythrocarpa

Levels of saponins and phenolic compounds (including coumarins, flavonols, catechins, tannins, and phenolic acids) were studied in extracts from the above-ground and underground parts of the two species of *Actaea* plants (Table 1). Higher concentrations of most of the analyzed biologically active substances were found in *A. erythrocarpa* compared to *A. cimicifuga*: in the extract from the *A. erythrocarpa* above-ground part, levels of phenolic compounds (101.3 mg/g of DE) [including catechins (10.9 mg/g of DE), coumarins (161.6 mg/g of DE), and flavonols (32.42 mg/g of DE)] and levels of saponins (247.9 mg/g of DE) proved to be the highest among the four extracts; levels of tannins (202.3 mg/g of DE) and phenolic acids (112.2 mg/g of DE) were the highest in the extract from rhizomes and the roots of *A. erythrocarpa*.

In general, it was noted that extracts from the above-ground part of both *Actaea* species exhibited a higher concentration of phenolic compounds (including coumarins, catechins, and flavonols) and saponins compared to extracts from the underground part. In turn, extracts from rhizomes and roots were found to be rich in phenolic acids. The pattern of tannins’ distribution differed between the two *Actaea* species in question: within *A. erythrocarpa*, these substances were found to be more abundant in the underground part, whereas their levels in *A. cimicifuga* were comparable between the above-ground and underground parts.

### 2.2. Metabolites (In Extracts from Actaea Species) Identified by LC-HRMS

Chromatographic profiles of the above-ground and underground parts of *A. erythrocarpa* and *A. cimicifuga* were obtained by LC-HRMS. The metabolites of both species were identified using retention times, and a comparison with the mass spectra of standards and data from the databases mzCloud, mzVault, and ChemSpider was conducted. More than 60 compounds were isolated and identified in the extracts from *A. erythrocarpa* and *A. cimicifuga* (Table 2).

In the analyzed extracts from *A. erythrocarpa* and *A. cimicifuga*, a wide variety of cinnamic-acid derivatives (compounds **1**–**13**) was found, including caffeic (**2**), ferulic (**11**), and isoferulic (**12**) acids. The simultaneous presence of ferulic and isoferulic acids in extracts is rare among plants, and the presence of their condensation products called cimicifugic acids is considered species-specific [13]. From this subclass, in *A. erythrocarpa* and *A. cimicifuga*, cimicifugic acids A (**9**) and B (**10**) were found. The previous analyses of the antioxidant activity of extracts from *Actaea* species enriched with this subclass of compounds have revealed their strong ability to scavenge free radicals [18,19,20]. It was also reported that this antioxidant activity is dependent on the number of hydroxyl groups in phenolic acids and their derivatives [19].

Flavonoids are not the most diverse class of compounds in *Actaea* species; in earlier research articles, flavones and isoflavones have been mentioned the most often [13]. We also identified several flavones: zapotin (**14**), chrysin (**15**), 4′,5,7-trihydroxy-3-methoxyflavone-3′-*O*-xylopyranoside (**16**), and an isoflavone referred to as 5-methoxy-7,2′,4′-trihydroxyisoflavone (**17**). In addition, quercetin (**18**), kaempferol (**22**), and their derivatives (**19**–**21, 23, 24**) were identified.

The most prevalent subclass of metabolites was triterpenes: compounds based on three terpene units that are often cyclized into systems of 1–5 rings. To date, more than 200 triterpene compounds have been identified in *Actaea* species, with the most diverse subclass being tetracyclic triterpenoids, conventionally categorized into seven subtypes [13]. Oleanolic (**25**), asiatic (**26**), 7-hydroxy-betulonic (**30**), and 18-β-glycyrrhetinic (**29**) acids, which were found in the extracts from *A. erythrocarpa* and *A. cimicifuga,* are common secondary metabolites known for their antitumor/antileukemic, anti-inflammatory, antimicrobial, antioxidant, and other activities [21,22,23,24]. A sesquiterpene called zerumbone (**33**) is used in medicine due to its anticancer and antimicrobial properties [25]. It is worth mentioning that 23-epi-26-deoxyactein (**28**) has so far only been found in *Actaea* species. The concentration of this metabolite can constitute up to 15% of the total triterpene content, and its presence in extracts from individual species can serve as a chemical marker of the authenticity of such plant products [26]. Extracts consisting mainly of 23-epi-26-deoxyactein are nontoxic and are alternatively employed as a form of hormonal therapy in women with menopausal symptoms [27].

Various alkaloid glycosides, amide derivatives, and other nitrogenous compounds are also characteristic of *Actaea* species [15]. Little attention has been given to this class, although more than 70 nitrogenous compounds have been isolated from and identified in *A. racemosa* extracts alone, many of which are not only specific to *Actaea* species but have also been discovered in plants for the first time [28]. At least 10 such compounds were detected in our extracts. The detected nitrogenous bases of thymine (**38**) and guanine (**41**), the vitamin-like compound choline (**39**), and DL-glutamic acid (**40**) are primary metabolites and are present in most known living organisms. Of note, salsolinol (**37**) was found in all the extracts analyzed in the present study. This alkaloid has been repeatedly recorded in *A. racemosa* [29,30], and it is believed that salsolinol has a strong effect on the central nervous system and is associated with the observed in vitro dopaminergic activity of extracts from *A. racemosa* [31]. Nitrogenous compounds of *A. erythrocarpa* and *A. cimicifuga* require further, in-depth research as they are undoubtedly biologically active substances.

Furthermore, a number of fatty acids and their derivatives were identified in our extracts (**54**–**66**). Nine compounds were identified as coumarins: **42**–**46** are simple coumarins with hydroxyl and methyl substituents, **47** and **50** are coumarin glycosides, **48** is a coumarin carrying a substituent in the form of a geraniol residue, and **49** is a furocoumarin.

Chromones are represented by three compounds (**51**–**53**). An interesting feature associated with the chromone called cimifugin was noted in the study by Jang et al. [32]: here, cimifugin was detectable in assayed Asian *Actaea* species and was not identified in American ones. This specificity could serve as a chemical marker; however, a different assay detected cimifugin in extracts of all these species. Thus, it should be noted that this compound may be undetectable when using different sample preparation methods [32]. In our work, all three chromones were recorded in both species under study.

### 2.3. HPLC Analysis of the Content of Individual Compounds in Extracts from Actaea Species

In the extracts from the above-ground and underground parts of *A. cimicifuga* and *A. erythrocarpa*, substances were quantified by HPLC by means of standard compounds (Table 3). In the extracts from the above-ground parts of the species, caffeic, ferulic, and isoferulic acids, hyperoside, quercetin, and kaempferol were detected; additionally, benzoic acid was found in the extract from *A. erythrocarpa*, and kaempferol was found in the extract from *A. cimicifuga*.

In the extracts from the roots and rhizomes of the two *Actaea* species, only acids were identified, while flavonols were detectable only in trace amounts. It was noted that among the four extracts, the concentration of caffeic acid (0.74 mg/g of DE) is the highest in the extract from the *A. erythrocarpa* underground part, whereas the extract from the above-ground part of this species contains the highest levels of ferulic (1.16 mg/g of DE) and isoferulic acids (1.49 mg/g of DE) and of hyperoside (13.05 mg/g of DE). The concentration of free quercetin was the highest (1.58 mg/g of DE) in the extract from the above-ground part of *A. cimicifuga*.

In hydrolysates of the extracts from the two *Actaea* species, aglycones of flavonols (quercetin, kaempferol, and isorhamnetin) were detected (Table 3). Furthermore, the aglycones of flavonols were identified only in extracts from the above-ground part, while in extracts from the roots and rhizomes, only traces of these compounds were recorded. All the identified flavonol aglycones had the highest concentrations in the extract from the above-ground parts of *A. erythrocarpa*.

### 2.4. Cytotoxicity of Extracts from A. cimicifuga and A. erythrocarpa

This study showed that the extract from the above-ground part of *A. erythrocarpa* exhibited a more pronounced cytotoxic activity against 3T3-L1 and MDA-MB-231 cells (Table 4). The extracts from the above-ground parts of both plants showed cytotoxic activity against HepG2 tumor cells at similar concentrations (236 and 264 μg/mL), while the extracts from the roots and rhizomes suppressed the growth of HepG2 cells at higher concentrations. The extracts from the above-ground part also showed a more toxic effect on the 3T3-L1 cell line. The extract from the roots and rhizomes of *A. cimicifuga* showed cytotoxic activity at the studied concentrations against 3T3-L1 cells only. The lack of cytotoxic activity against tumor cells may be attributed to the lower content of phenolic compounds, flavonoids, and terpenoids in the extract from the underground part compared to that from the above-ground part.

The results of the present study are comparable and confirmed by the work of other researchers. Thus, Shi Q. Q. et al. [33] showed that triterpenoid–chromone hybrids, isolated for the first time from *A. cimicifuga* rhizomes, exert cytotoxic activity against taxol-resistant human lung cancer A-549/taxol, which is comparable with the value of the positive control cisplatin. Tetracyclic terpenoids actein and 26-deoxyactein, isolated from the rhizome of *A. cimicifuga*, exhibited pronounced cytotoxic activity in experiments on 12 cancer cell lines. In vivo studies showed that these terpenoids cause the dose-dependent inhibition of the growth of sarcoma S180 implanted in mice. The authors attributed the antitumor activity to the inhibition of the cell cycle and angiogenesis [34]. Cycloartane terpenoids, namely cimigenol and 23-epi-26-deoxyactein, isolated from the roots of *A. yunnanensis*, demonstrated significant inhibitory activity against the breast cancer cell lines MDA-MB-231, MCF-7, and SK-BR3 [35]. The antiproliferative activity of cimicifugic acids A and B, which are part of the extract from the rhizome of *Cimicifuga heracleifolia* (=*A. heracleifolia*), against the human colon cancer cell line (HCT116), was established [36].

### 2.5. Antioxidant Activity of Extracts from A. cimicifuga and A. erythrocarpa

The antioxidant activity of extracts from species of the genus *Actaea* has been reported in numerous studies. For instance, a crude methanol extract from *A. racemosa* rhizomes showed DPPH radical-scavenging activity with an IC_50_ of 99 μg/mL compared to the positive control with gallic acid (IC_50_ = 4.92 μg/mL) [37,38]. The analysis of the antioxidant activity of four North American *Actaea* species showed that the extract from *A. rubra* (IC_50_ = 79.3 μg/mL) exhibited superior DPPH radical-scavenging activity compared to the extracts from *A. pachypoda* (IC_50_ = 191.6 μg/mL), *A. podocarpa* (IC_50_ = 111.1 μg/mL), and *A. racemosa* (IC_50_ = 144.6 μg/mL) [39]. The extracts with different polarity from the rhizome of *Actaea* exhibited antioxidant activity in the DPPH test with IC_50_ values in the range of 227–813 μg/mL. The most significant activity was demonstrated by ethyl acetate and aqueous extracts from rhizomes [18]. The analysis of the antioxidant activity of the studied species of the genus *Actaea* also showed high antioxidant potential.

**Figure 1:**
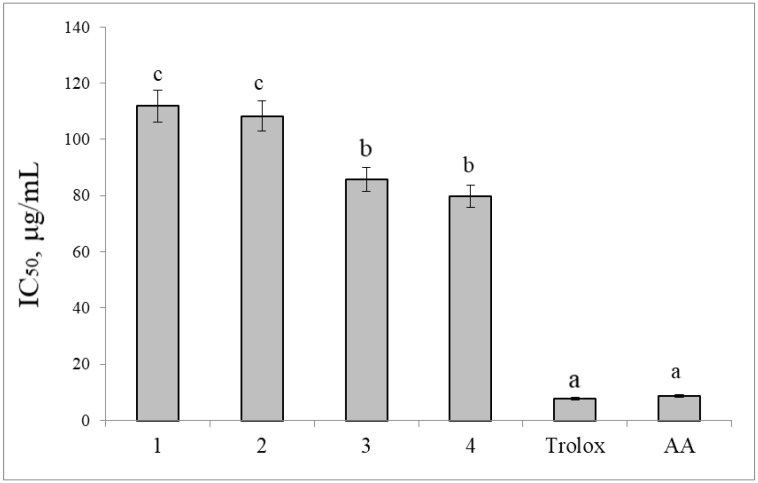
DPPH radical-scavenging activity of the extracts. IC_50_, μg/mL: the antioxidant concentration that causes 50% DPPH loss in the assay of DPPH radical-scavenging activity. Different letters indicate significant differences in this parameter (*p* ≤ 0.05) according to Tukey’s HSD test. Definitions: 1: the above-ground parts of *A. cimicifuga*; 2: the underground part of *A. cimicifuga*; 3: the above-ground parts of *A. erythrocarpa*; 4: the underground part of *A. erythrocarpa*; AA: ascorbic acid.

The assay of antioxidant activity in hydroethanolic extracts from *Actaea* plants revealed that *A. erythrocarpa* extracts have a higher antioxidant activity [Figure 1]. In addition, the extract from the underground part of *A. erythrocarpa* (IC_50_ = 79.7 μg/mL) proved to be a slightly more promising antioxidant compared to the extract from the above-ground part (IC_50_ = 85.8 μg/mL). The high antioxidant activity of the root-and-rhizome extracts can be attributed to their higher levels of tannins and phenolic acids, and the high antioxidant activity of extracts from the above-ground part of *A. erythrocarpa* may be due to the substantial presence of phenolic compounds (including catechins, coumarins, and flavonols) and saponins. The preliminary data obtained on the direct correlation between biological activity and the concentration of phenolic compounds require additional statistical analysis to be performed in future studies.

In the DPPH assay, the antioxidant activity did not differ significantly between the extracts from the above-ground part (IC_50_ = 111.8 μg/mL) and the underground part (IC_50_ = 108.3 μg/mL) of *A. cimicifuga*. It should be noted that the data obtained on the ability of the *A. erythrocarpa* extract to scavenge the DPPH radical correlates with its cytotoxicity against the studied cell lines.

## 3. Materials and Methods

### 3.1. Plant Material

*Actaea* plant material was collected from natural populations in Siberia: *A. cimicifuga* was found in the Republic of Khakassia (Russia), Bogradsky district, in the vicinity of the Bograd village; 54°14′28.4″ N, 90°46′26.1″ E 544 m above sea level; in a birch forest; 19 August 2023. *A. erythrocarpa* was located in Krasnoyarsk Krai (Russia), Minusinsk district, in the vicinity of the Znamenka village; 53°32′43.4″ N, 91°56′11.1″ E, 461 m above sea level, 15 July 2023. Voucher (*A. cimicifuga*–No. NS0033873; *A. erythrocarpa*–No. NS0033874) specimens were placed in the Plant Material Storage Room of the Laboratory of Herbarium of the Central Siberian Botanical Garden SB RAS (Novosibirsk, Russia). The plants were harvested at the flowering stage. Extracts were prepared from the above-ground and underground parts (roots and rhizomes) of *A. cimicifuga* and *A. erythrocarpa*. Air-dry plant material was ground in an A11 basic mill (IKA, Staufen im Breisgau, Germany) and then sieved using a 5 mm sieve.

### 3.2. Ethanol Extract Preparation

Each fluid extract was prepared in glass diffusers using the method of three-stage countercurrent repercolation with a complete cycle in the ratio of 1:1 for the raw materials compared to the resulting product [40]. The extractant used was a 40% ethanol solution.

The resulting fluid extract was subjected to a drying process using a dry evaporation vacuum system (Labconco, RapidVap, Kansas City, MO, USA) at 35 °C under pressure that did not exceed 50 mBar. The residual moisture content of the obtained extracts was 2.9–3.8%.

The antioxidant activity and biologically active substances present in the *Actaea* extracts were studied via the dissolution of the thick extract (0.1 g) in 40% ethanol (10 mL). For LC-HRMS and HPLC analyses, 1 mL of extract was diluted with double-distilled water to 5 mL and passed through a Diapak C16 concentrating cartridge (BioKhimMak Co., Moscow, Russia). Substances were washed off the cartridge with a small amount (3 mL) of 40% ethanol and then with 96% ethanol (2 mL). The combined eluate was filtered through a membrane filter with a pore diameter of 0.45 μm.

### 3.3. Quantification of Biologically Active Substances in Actaea Extracts

The total phenolic compounds level was assessed using the Folin–Ciocalteu reagent (Sigma, St. Louis, MO, USA) [41]. Absorbance was measured at a wavelength of 765 nm using an SF-56 spectrophotometer (Lomo, St. Petersburg, Russia). The standard curve was constructed with gallic acid (concentration of 0.002–0.010 mg/mL; the calibration curve equation is as follows: y = 116.15x + 0.0256 (R^2^ = 0.9971)), and the total phenolic content was expressed as mg of gallic acid (Sigma-Aldrich, Saint Louis, MO, USA) equivalent per gram of the dry extract (DE) using the standard curve.

Flavonol contents were determined by a spectrophotometric method that involved the complex formation reaction between flavonols and aluminum chloride [42]. The optical density of the solution with aluminum chloride was determined using the SF-56 spectrophotometer (Lomo, St. Petersburg, Russia) at 415 nm in a cuvette with a 1 cm light path. A solution of acetic acid was used as the control. The flavonol level in the sample was assessed using the calibration curve, which was built based on the calibration curve for rutine (Chemapol, Mumbai, MH, India). At concentrations of 0.002–0.010 mg/mL, the following calibration curve equation was produced: y = 25.1x + 0.4572 (R^2^ = 0.9574). The results obtained were expressed as mg of the rutin equivalent per gram of DE using the standard curve.

The catechin concentration was assessed through spectrophotometric analysis using a method that relies on the ability of catechins to create a crimson color in a solution of vanillin and concentrated hydrochloric acid [43]. The color intensity was measured using the SF-56 spectrophotometer (Lomo, St. Petersburg, Russia) at 504 nm in a cuvette with a 1 cm light path. The standard curve was built with (±)-catechin (Sigma, St. Louis, MO, USA) at concentrations of 0.001–0.020 mg/mL. The catechin content was expressed as mg of a (±)-catechin equivalent per gram of DE based on the standard curve.

Tannins (hydrolyzable tannins) were quantified using the method by L.M. Fedoseeva [44]. The resulting color intensity was measured using the SF-56 spectrophotometer (Lomo, St. Petersburg, Russia) at 420 nm in a cuvette with a 1 cm light path. A government-standard sample of tannin (Sigma, St. Louis, MO, USA, concentration of 0.002–0.010 mg/mL; the calibration curve equation is the following: y = 5.7205x + 0.0144 (R^2^ = 0.999)) served as the standard. The results were expressed as mg of the tannin equivalent per gram of DE using the standard curve.

The total phenolic acid content was determined using Arnov’s reagent [45,46]. Optical density was determined immediately at 490 nm using the SF-56 spectrophotometer (Lomo, St. Petersburg, Russia). The obtained results were expressed as mg of caffeic acid (Serva, Heidelberg, Germany, concentration of 0.02–0.10 mg/mL; the calibration curve equation is the following: y = 1.5786x − 0.1314 (R^2^ = 0.9944)) with equivalents per gram of DE based on the standard curve.

A detailed description of the methods for quantifying phenolic compounds, including flavonol, catechins, tannins, and phenolic acids, is given in refs. [47,48].

The total content of saponins was quantified using a direct spectrophotometric method that involved a reaction between triterpene saponins and sulfuric acid [49]. A precise amount of DE (0.5–1.0 g) was dissolved in 10 mL of the mixture composed of glacial acetic acid, hydrochloric acid, and purified water (3.5:1.0:5.5) to hydrolyze glycosides via 2 h of incubation in a boiling water bath. Next, the hydrolysate was cooled, then diluted with distilled water in a 1:2 ratio, and filtered. The residue on the filter was first rinsed with distilled water and then placed with the filter into a volumetric 25 mL flask. It was then dissolved in 25 mL of hot ethanol. A volume of 4 mL of concentrated sulfuric acid was added to 1 mL of this ethanolic solution. Absorbance at 310 nm was measured after 10 min with the SF-56 spectrophotometer (Lomo, St. Petersburg, Russia). Concentrated sulfuric acid was used as a blank control. A calibration curve for oleanolic acid built in the range of 0.01–0.40 mg/mL was used to calculate the saponin concentration; the calibration curve equation is as follows: y = 5.1963x + 0.0276 (R^2^ = 0.9477). The data obtained were expressed as milligrams of an oleanolic acid equivalent per gram of DE.

For the quantification of coumarins, the method by De Amorim et al. [50] was utilized. A total of 500 μL of the extract was transferred to a test tube, and 2 mL of distilled water and 500 μL of a 5% lead acetate solution were introduced. The sample was shaken, 7 mL of distilled water was added, and 2 mL of the solution was poured into a new test tube. Then, 8 mL of a 0.1 M hydrochloric acid solution was added. The samples were incubated at room temperature for 30 min. Measurements were carried out using the SF-56 spectrophotometer at 320 nm in a cuvette with a 1 cm light path (Lomo, St. Petersburg, Russia). The concentration of coumarins in each sample was determined using the calibration curve for coumarin (Sigma, St. Louis, MO, USA); the calibration curve equation is as follows: y = 35.385x – 0.016 (R^2^ = 0.9932).

### 3.4. Liquid Chromatography Coupled with High-Resolution Mass Spectrometry (LC-HRMS) Analysis of Metabolites in Actaea Extracts

LC-HRMS was conducted at the Core Facility of Mass-Spectrometric Analysis at the Institute of Chemical Biology and Fundamental Medicine SB RAS (Novosibirsk, Russia). An Ultimate 3000 liquid chromatograph (Thermo Fisher Scientific, San Jose, CA, USA) equipped with a Q Exactive HF mass spectrometer (Thermo Fisher Scientific, San Jose, CA, USA) was used to determine the metabolomic profiles of *Actaea* extracts. Chromatographic separation was performed at a 0.4 mL/min flow rate on a Hypersil GOLD aA reversed-phase column (100 mm × 2.1 mm, 1.9 μm, Thermo Fisher Scientific, Santa Clara, CA, USA) thermostatted at 40 °C. The mobile phase comprised 0.1% aqueous formic acid (eluent A) and 0.1% formic acid in acetonitrile (eluent B). The elution gradient increased from 5% to 70% B within the first 40 min, followed by its growth to 90% B within 8 min before decreasing to 5% B within 5 min and becoming re-equilibrated under the initial conditions within 7 min. The electrospray ionization (ESI) source was set as follows: an electrospray of 3.2 kV in the negative mode and 4.2 kV in the positive mode; a capillary temperature of 350 °C; and an S lens RF level of 50. Two methods were used to obtain the data. A full scan was used to detect the compounds, and full-scan data-dependent acquisition (FS-dd-MS2) was used to identify them. To detect the compounds, scanning was conducted in positive and negative modes at a resolving power of 120,000 full width at half maximum (FWHM) over *m*/*z* 200. The mass spectrometer was set as follows: scan range of *m*/*z* 100–1000; automatic gain control (AGC) of 1 × 10^6^; and maximum accumulation ion time (Maximum IT) of 100 ms. Compounds were identified using positive and negative scans at a resolving power of 45,000 FWHM over *m*/*z* 200. The mass spectrometer was set as follows: scan range of *m*/*z* 100–1000; AGC of 3 × 10^6^; and Maximum IT of 100 ms. The targeted tandem mass spectrometry (MS/MS, i.e., dd-MS2) analysis was conducted in positive and negative modes at 30,000 FWHM (*m*/*z* 200) with an isolation window of *m*/*z* 1.0. The normalized collision energy for molecular ion fragmentation was set to 20, 40, and 60 eV. The value of the AGC for dd-MS2 was 1 × 10^5^, with a Maximum IT of 100 ms and a loop count of 10. In the “dd settings” section, the AGC target was set to 8 × 10^3^. The data analysis was performed using Xcalibur 4.2 and Compound Discoverer 3.2 software (Thermo Fisher Scientific, San Jose, CA, USA). All the samples, including blank samples, were assayed in triplicate. The processing of all the samples was performed using Compound Discoverer 3.2 via a common workflow: “Environmental Unknown ID w Online and Local Database Searches.” A mass tolerance of 10 ppm was applied to all nodes. Several databases, i.e., KEGG [51], MassBank [52], PlantCyc [53], Planta Piloto de Quimica Fina Universidad de Alcala [54], AraCyc [55], Extrasynthese [56], Indofine [57], and Sequoia Research Products [58] were chosen in ChemSpider. A more detailed procedure for substance identification is described in [59].

A number of mzVault libraries were used in the mzVault Search node: Creation of a PlantMetabolite Spectral Library for Untargeted and targetedMetabolomics.db [60], Negative ionmode_Jan2021.db, Positive ion mode_Jan2021.db [61], and MS2_library.db [62]. Metabolites were identified using both accurate mass and fragment mass “fingerprint” spectra via searches against the spectra of compounds available in the mzCloud database [63] and mzVault. Some metabolites were identified using standards. If mzCloud and mzVault lacked compounds, they were tentatively identified using the ChemSpider search. According to the workflow, the masses extracted from the chromatograms were aligned and filtered to remove (i) background compounds present in the blank sample and (ii) masses of compounds that were absent from the databases.

### 3.5. Analysis of Metabolite Levels in Extracts from Actaea Plants Using High-Performance Liquid Chromatography (HPLC)

Phenolic compounds in each eluate were analyzed on an analytical HPLC system that comprises an Agilent 1200 liquid chromatograph (Santa Clara, CA, USA) with a diode array detector, an autosampler, and a ChemStation system for collecting and processing chromatographic data.

The substances were separated using a Diasfer-110-C18 column (BioKhimMak Co., Moscow, Russia) (4.6 × 150 mm in size, with a particle diameter of 5 μm) via gradient elution. For extract analysis, the system was used with a level of methanol applied in an aqueous solution of orthophosphoric acid (0.1%) in the mobile phase that varied from 19% to 70% during 30 min, then up to 100% at minute 32. The eluent flow rate was 1 mL/min. The column temperature was 25 °C, and the volume of the injected sample was 10 μL. Detection was performed at analytical wavelengths of λ = 255, 270, 290, 325, 340, 350, 360, and 370 nm. Methanol (special purity grade), orthophosphoric acid (special purity grade), and double-distilled water were used to prepare the mobile phases. The quantification of phenolic compounds was performed as reported previously [64]. To prepare the standard samples, gentisic and cinnamic acids (Serva Heidelberg, Germany), chlorogenic and caffeic acids, quercetin, kaempferol, nicotiflorin, orientin, isorhamnetin-3-rutinoside, quercetin-3-glucuronoside, coumarin, luteolin-7-glucoside (Sigma-Aldrich, St. Louis, MO, USA), ferulic acid, isoquercitrin, avicularin, astragalin, rutin, spiraeoside, hyperoside, vitexin, and quercitrin (Fluka, Everett, WA, USA) were used. Standard solutions were prepared at a concentration of 10 µg/mL.

### 3.6. Cytotoxicity Assay

The cell lines of noncancerous mouse fibroblasts (3T3-L1) and epithelial liver cancer (HepG2) were obtained from Vector (Koltsovo, Russia). Human mammary gland cancer (MDA-MB-231) cells were obtained from ECACC (Salisbury, UK). Cell culturing was performed in Dulbecco’s modified Eagle’s medium/F12 (Sigma-Aldrich, USA) with the following supplements: 10% fetal bovine serum (FBS) (Global Kang Technology, Qinhuangdao, China), 1% GlutaMAX glutamic acid solution (Gibco, Waltham, MA, USA), and 1% antibiotic/antimycotic (Gibco, USA). The cells were cultured in a humid atmosphere with 5% CO_2_ at 37 °C. To evaluate the cytotoxicity of the *A. erythrocarpa* and *A. cimicifuga* extracts from the above-ground and underground parts, a slightly modified 3-(4,5-methylthiazol-2-yl)-2,5-diphenyl tetrazolium bromide (MTT) assay was used. This technique employs the ability of mitochondrial dehydrogenases in a live cell to reduce waSSSter-soluble MTT into formazan crystals. The cells were seeded in 96-well plates (SPL Life Sciences, Korea) at 10,000 per well and then incubated in a CO_2_ incubator (Sanyo, Japan) for 24 h to allow adhesion and 80–90% confluence. The following day, stock solutions of the extracts in DMSO (Scharlab S.L., Sentmenat, Spain) and serial twofold dilutions were prepared. Concentrations of the sample from the *A. erythrocarpa* above-ground part were 500.00–3.91 μg/mL, and those of the sample from its roots were 1000.0–7.8 μg/mL; concentrations of the samples from the *A. cimicifuga* above-ground part and roots were 1000.0–7.8 μg/mL. A volume of 1 μL of this solution was added to the wells of a 96-well plate with cells, while the DMSO concentration was 0.5%. The equivalent DMSO volume (without an extract) was added to control cells. Triton X100 (Merk, Rahway, NJ, USA) was used as the positive control.

After the addition of the tested extracts, the plates with cells were kept in a CO_2_ incubator for 24 h. After that, the medium in the wells was replaced with a fresh one, and 10 μL of the MTT reagent (5 mg/mL) (NeoFroxx GmbH, Einhausen, Germany) dissolved in sterile PBS (Merk, USA) was introduced into each well. After 2 h of incubation, the medium was removed from the wells, and 200 µL of DMSO was added into each well to dissolve the formazan crystals. The absorbance of the resultant solutions was measured in each well at 540 nm and at a reference wavelength of 650 nm on an Infinite M Plex automated microplate analyzer with a Te-Inject system (Tecan Austria GmbH, Grödig, Austria). The concentration of the extract was calculated at which the viability of the cell culture was reduced to 50% of the control (IC_50_).

### 3.7. Antioxidant Activity Assay Using 2,2-Diphenyl-1-Picrylhydrazyl (DPPH)

The free radical-scavenging ability of the extracts was assessed using the DPPH method [50,59]. For this purpose, a 2 mL aliquot of the extract (the extracts were diluted with 40% ethanol to concentrations in the range of 20–1200 µg/mL) was mixed with 3 mL of the DPPH solution (62 µg/mL in ethanol). After 40 min of incubation in the dark at room temperature, absorbance (D) was measured at 517 nm against the blank. The results are expressed in terms of DPPH IC_50_, and the concentration of the antioxidant required to cause a 50% loss of DPPH in the DPPH radical-scavenging activity assay was ascertained. Solutions of 6-hydroxy-2,5,7,8-tetramethylchroman-2-carboxylic acid [Trolox] and ascorbic acid (concentrations of 2.5–50.0 μg/mL) served as a positive control. A more detailed description of the method is given in [65,66].

### 3.8. Statistical Analysis

The analysis was conducted using GraphPad Prism v8.4.3 (San Diego, USA) and Microsoft Excel 2016. The antioxidant activity and total phenolic content were assayed in three technical replicates.

## 4. Conclusions

To conclude, the above-ground and underground parts of *A. cimicifuga* and *A. erythrocarpa* are natural accumulators of biologically active substances. Using LC-HRMS, their metabolites were separated and identified (more than 60 compounds), including phenolic compounds, nitrogenous compounds, chromones, and triterpenes. Most secondary metabolites have been reported for these species for the first time. The HPLC analysis was employed to determine the concentrations of some of these substances in extracts from the above-ground and underground parts of the species. The diversity of secondary metabolites in the above-ground and underground parts of *A. cimicifuga* and *A. erythrocarpa* suggests the presence of biological activity, which was confirmed by in vitro analyses. The results indicate more pronounced antioxidant and antitumor activities in *A. erythrocarpa* compared to *A. cimicifuga*. The information obtained suggests that the species studied could have potential as sources of medicinal products; however, further pharmacological studies of *A. cimicifuga* and *A. erythrocarpa* extracts are required to determine the prospects for their therapeutic and prophylactic use.

## Figures and Tables

**Table 1:** Levels of biologically active substances in extracts from *A. cimicifuga* and *A. erythrocarpa*.

Substance Level, mg/g	Species (Plant Part)			
	*A. cimicifuga* (Above-Ground Parts)	*A. cimicifuga* (Roots and Rhizomes)	*A. erythrocarpa* (Above-Ground Parts)	*A. erythrocarpa* (Roots and Rhizomes)
Phenolic compounds	79.31 ± 3.15 ^b^	67.10 ± 2.66 ^c^	101.31 ± 4.02 ^a^	79.19 ± 3.14 ^b^
Catechins	7.04 ± 0.12 ^b^	3.55 ± 0.06 ^d^	10.92 ± 0.19 ^a^	5.04 ± 0.09 ^c^
Flavonols	20.04 ± 0.60 ^b^	8.08 ± 0.24 ^c^	32.42 ± 0.97 ^a^	8.12 ± 0.24 ^c^
Tannins	149.51 ± 1.27 ^b^	130.26 ± 1.11 ^c^	147.83 ± 1.26 ^b^	202.31 ± 1.72 ^a^
Phenolic acids	71.27 ± 2.85 ^d^	95.15 ± 3.81 ^b,c^	104.48 ± 4.18 ^a,b^	112.18 ± 4.49 ^a^
Saponins	178.90 ± 0.02 ^c^	154.34 ± 0.06 ^d^	247.88 ± 0.06 ^a^	225.86 ± 4.95 ^b^
Coumarins	92.76 ± 3.71 ^c^	70.47 ± 2.82 ^d^	161.55 ± 6.46 ^a^	105.44 ± 4.22 ^b^

Note: means followed by different letters in the same row are significantly different (*p* ≤ 0.05) according to Tukey’s HSD test.

**Table 2:** Metabolites tentatively identified in hydroethanolic extracts from *Actaea* species by LC-HRMS using the databases mzCloud, mzVault, and ChemSpider.

Compound Number	Identified Compounds	t_R_ (min)	Calculated Mass (Da)	Found Mass	Delta of Mass (ppm)	Score	Mode	*A. cimicifuga*		*A. erythrocarpa*	
								AP	RR	AP	RR
**Phenolic Compounds**											
**1**	Benzoic acid *	3.20	122.037	122.037	2.01	–	p	–	–	+	+
**2**	Caffeic acid *	10.08	180.042	180.042	0.76	96.2 ^1^	p	+	+	+	+
**3**	Methyl caffeate	8.73	194.058	194.058	0.75	–	p	+	+	+	+
**4**	2-Hydroxycinnamic acid	12.81	164.047	164.048	1.32	75.9 ^1^	p	+	+	+	+
**5**	3,4-Dimethoxycinnamic acid	12.62	208.074	208.077	15.81	88.8 ^1^	p	+	+	+	+
**6**	2-Hydroxy-4-methoxycinnamic acid	16.89	194.058	194.058	1	81.7 ^1^	p	+	+	+	+
**7**	2,5-Dihydroxycinnamic acid	3.55	180.042	180.042	0.16	71.8 ^1^	p	–	–	+	+
**8**	4-Methoxy-cinnamic acid-2-glucoside	7.29	356.111	356.111	0.66	78.4 ^1^	P	+	+	–	–
**9**	Cimicifugic acid A	10.44	448.101	448.101	0.14	65.6 ^1^	p	+	+	+	+
**10**	Cimicifugic acid B	13.62	448.101	448.092	−18.75	93.7 ^1^	n	+	+	+	+
**11**	Ferulic acid *	12.03	194.058	194.058	1.29	90.5 ^2^	p	+	+	+	+
**12**	Isoferulic acid *	11.73	194.058	194.058	0.6	91.7 ^1^	p	+	+	+	+
**13**	*p*-Coumaric acid *	14.31	164.047	164.048	1.31	90.3 ^1^	p	+	+	+	+
Flavonoids											
**14**	Zapotin	0.31	342.110	342.116	17.44	57 ^1^	p	–	–	+	+
**15**	Chrysin	11.19	254.058	254.058	0.57	75.6 ^1^	p	–	–	+	+
**16**	4′,5,7-Trihydroxy-3-methoxyflavone-3′-O-xylopyranoside	10.70	448.101	448.100	−0.25	53.5 ^1^	p	–	–	+	+
**17**	5-Methoxy-7,2’,4’-trihydroxyisoflavone	19.97	300.063	300.058	−19.49	65.5 ^1^	n	+	+	–	–
**18**	Quercetin *	9.90	302.043	302.043	−0.04	93.9 ^1^	p	+	+	+	+
**19**	Isorhamnetin	10.78	316.058	316.058	0.04	95.4 ^1^	p	+	+	+	+
**20**	Hyperoside *	9.46	464.095	464.095	−0.08	93.9 ^1^	p	+	+	+	+
**21**	Quercetin-6-O-β-D-xylopyranosyl-β-D-glucopyranoside	8.57	596.138	596.137	−0.51	87.9 ^1^	p	+	+	+	+
**22**	Kaempferol *	9.61	286.048	286.048	0.14	89.6 ^1^	p	+	+	–	–
**23**	Trifolin	13.79	448.101	448.092	−18.97	61.1 ^1^	n	–	–	+	+
**24**	Kaempferol-3-O-xylopyranosyl-glucopyranoside	9.86	580.143	580.143	−0.21	79.4 ^1^	p	–	–	+	+
Terpenes											
**25**	Oleanolic acid	35.67	456.360	456.360	0.29	-	p	+	+	–	–
**26**	Asiatic acid	20.69	488.350	488.350	−0.27	62.2 ^1^	p	+	+	+	+
**27**	2,24-Dihydroxyursolic acid	33.54	488.350	488.350	0.08	65.3 ^1^	p	–	–	+	+
**28**	23-Epi-26-deoxyactein	23.56	660.387	660.387	−0.02	84.2 ^1^	P	+	+	+	+
**29**	18-β-Glycyrrhetinic acid	23.50	470.340	470.340	0.39	75.1 ^2^	p	+	+	+	+
**30**	7-Hydroxy-betulonic acid	26.31	470.340	470.340	0.22	66.5 ^1^	P	+	+	–	–
**31**	1-Oxo-3β,23-dihydroxyolean-12-en-28-oic acid	18.94	486.335	486.342	15.92	63.6 ^1^	p	–	–	+	+
**32**	2-Hydroxy-22-(2-hydroxy-2-propanyl)-3,8,8,17,19-pentamethyl-23,24-dioxaheptacyclo[19.2.1.01,18 03,17 04,14 07,12 012,14]tetracos-4-en-9-yl β-D-xylopyranoside	22.52	618.377	618.387	16.24	70.7 ^1^	p	+	+	+	+
**33**	Zerumbone	30.32	218.167	218.171	17.03	64.6 ^1^	p	–	–	+	+
Nitrogen-containing constituents											
**34**	1,3-Dimethyl-6-morpholino-1,2,3,4-tetrahydropyrimidine-2,4-dione	3.19	225.111	225.111	0.66	64.3 ^1^	P	+	+	–	–
**35**	5-Amino-3-(4-methoxyphenyl)-5-oxopentanoic acid	1.24	237.100	237.100	1.01	73.4 ^1^	p	+	+	–	–
**36**	1,3,4,10-Tetrahydro-9(2H)-acridinone	19.25	199.100	199.100	0.94	66.6 ^1^	P	+	+	+	+
**37**	Salsolinol	0.68	179.095	179.095	0.91	67.4 ^1^	p	+	+	+	+
**38**	Thymine	1.06	126.043	126.043	2.02	86.5 ^1^	p	+	+	+	+
**39**	Choline	0.29	103.100	103.100	3.37	86.1 ^1^	p	+	+	+	+
**40**	DL-Glutamic acid	0.40	147.053	147.053	1.41	70.7 ^2^	p	+	+	+	+
**41**	Guanine	0.63	151.049	151.050	0.59	89.2 ^1^	p	+	+	+	+
Coumarins											
**42**	Scopoletin	6.88	192.042	192.042	1.17	72.5 ^1^	p	+	+	+	+
**43**	Fraxetin	7.28	208.037	208.037	1.05	62.6 ^1^	p	+	+	–	–
**44**	7,8-Dihydroxy-4-methylcoumarin	9.96	192.042	192.043	1.44	77.3 ^1^	p	+	+	+	+
**45**	Umbelliferone	10.12	162.032	162.032	0.55	78.7 ^1^	p	–	–	+	+
**46**	Magnolioside	6.88	354.095	354.095	−0.14	71.6 ^1^	p	+	+	+	+
**47**	5-Methylcoumarin-4-β-glucoside	11.53	338.100	338.100	−0.19	60.2 ^1^	p	+	+	+	+
**48**	Ostruthin	27.17	298.157	298.154	−8.57	55 ^1^	n	+	+	+	+
**49**	Isobergapten	19.59	216.042	216.042	0.95	68.6 ^1^	p	+	+	–	–
**50**	5-Xylopyranosyl-glucopyranoside 7,4′,5′-trihydroxy-4-phenylcoumarin	12.96	580.143	580.132	−18.89	85.3 ^1^	n	+	+	+	+
Chromones											
**51**	Cimifugin	9.83	306.110	306.110	−0.63	87.5 ^1^	p	+	+	+	+
**52**	Visnagin	17.21	230.058	230.058	−0.23	71.5 ^1^	p	+	+	+	+
**53**	5-Hydroxy-7-(hydroxymethyl)-2-methyl-2-(5-oxotetrahydro-2-furanyl)-2,3-dihydro-4H-chromen-4-one	10.44	292.095	292.095	0.27	60.9 ^1^	p	+	+	+	+
Fatty acids and their derivatives											
**54**	(±)13-HODE	37.20	296.235	296.229	−19.69	86.8 ^1^	n	+	+	+	+
**55**	(9Z,12Z)-6,8-Dihydroxy-9,12-octadecadienoic acid	25.36	312.230	312.224	−19.37	66.8 ^1^	n	–	–	+	+
**56**	(±)9-HpODE	26.47	312.230	312.224	−19.4	90.5 ^1^	n	+	+	+	+
**57**	13(S)-HOTrE	33.30	294.219	294.214	−19.24	85.8 ^1^	n	+	+	+	+
**58**	Arachidonic acid	36.69	304.240	304.240	0.58	70.4 ^1^	p	–	–	+	+
**59**	Juniperic acid	39.56	272.235	272.230	−19.27	92.3 ^1^	n	+	+	+	+
**60**	Corchorifatty acid F	19.02	328.225	328.219	−19.13	91.7 ^1^	n	+	+	+	+
**61**	α-Eleostearic acid	31.67	278.225	278.225	0.46	93.9 ^1^	p	+	+	+	+
**62**	α-Linolenic acid	31.38	278.225	278.225	0.44	88.3 ^1^	p	–	–	+	+
**63**	9-Oxo-ODE	32.43	294.219	294.220	0.33	75.8 ^1^	p	+	+	+	+
**64**	9S,13R-12-Oxophytodienoic acid	22.70	292.204	292.204	0.38	90.3 ^1^	p	–	–	+	+
**65**	4-Oxododecanedioic acid	9.75	244.131	244.131	0.38	67 ^1^	p	–	–	+	+
**66**	12-Oxo phytodienoic acid	26.82	292.204	292.204	0.5	92.9 ^1^	p	+	+	+	+

Note. t_R_—retention time; AP—above-ground parts; RR—roots and rhizomes. Score databases: “–” ChemSpider only; ^1^ mzCloud; ^2^ mzVault; “+”: the substance was detected in the extract; ppm = 10^−6^. * The compound was confirmed by means of a standard substance. Mode: p—positive; n—negative.

**Table 3:** Characteristics and levels of phenolic compounds found in *Actaea* extracts (mg/g of dry extract [DE]).

Peak ID	Compound	t_R_, min	Spectral Data λ_max_, nm	*A. cimicifuga*		*A. erythrocarpa*	
				AP	RR	AP	RR
**Extracts (chromatography with solvent I)**							
1	caffeic acid	10.6	240, 298 sh., 325	0.61 ± 0.02 ^b^	0.41 ± 0.02 ^d^	0.51 ± 0.02 ^c^	0.74 ± 0.03 ^a^
2	ferulic acid	15.1	235, 295 sh., 320	1.02 ± 0.04 ^b^	1.12 ± 0.04 ^a,b^	1.16 ± 0.04 ^a^	1.10 ± 0.04 ^a,b^
3	isoferulic acid	16.2	235, 295 sh., 320	1.29 ± 0.05 ^b^	0.12 ± 0.00 ^d^	1.49 ± 0.06 ^a^	0.54 ± 0.02 ^c^
4	benzoic acid	17.8	275	traces	traces	0.57 ± 0.02 ^a^	traces
5	hyperoside	18.6	255, 268 sh., 355	10.52 ± 0.39 ^b^	traces	13.05 ± 0.48 ^a^	traces
6	quercetin	24.5	255, 370	1.58 ± 0.06 ^a^	traces	1.05 ± 0.04 ^b^	traces
7	kaempferol	27.8	265, 365	0.05 ± 0.00 ^b^	traces	traces	traces
**Hydrolysate of extracts (chromatography with solvent II)**							
1	quercetin	9.4	255, 372	2.13 ± 0.08	traces	5.31 ± 0.20	traces
2	kaempferol	16.2	265, 365	1.12 ± 0.04	traces	2.38 ± 0.09	traces
3	isorhamnetin	18.0	255, 370	0.39 ± 0.01	traces	0.98 ± 0.04	traces

Note. t_R_—retention time; AP—above-ground parts; RR—roots and rhizomes; sh.: shoulder. Means followed by different letters in the same row are significantly different (*p* ≤ 0.05) according to Tukey’s HSD test.

**Table 4:** Cytotoxic activity of hydroethanolic *Actaea* extracts.

Species (Plant Part)	IC_50_ Toward Cell Lines µg/mL		
	3T3-L1	HepG2	MDA-MB-231
*A. erythrocarpa* (above-ground parts)	81 ± 3	236 ± 6	61 ± 5
*A. erythrocarpa* (roots and rhizomes)	728 ± 8	503 ± 3	554 ± 32
*A. cimicifuga* (above-ground parts)	230 ± 23	264 ± 29	782 ± 87
*A. cimicifuga* (roots and rhizomes)	711 ± 44	1023 ± 131	н/a

Note: the table shows the mean ± standard error (n = 3). 3T3-L1: a cell line of noncancerous mouse fibroblasts; MDA-MB-231: a human breast cancer cell line; HepG2: a human liver epithelial cancer cell line.

## Data Availability

Raw data of this study are available upon request from the corresponding author.
